# Development of a 3D in vitro model to study corpus luteum of felids based on luteinized cells from antral follicles

**DOI:** 10.1007/s00441-024-03937-z

**Published:** 2024-12-19

**Authors:** Michał M. Hryciuk, Filip Schröter, Svenja Claaßen, Christine Aurich, Jella Wauters, Celina Haße, Beate C. Braun

**Affiliations:** 1 Department of Reproduction Biology, Leibniz Institute for Zoo and Wildlife Research, Alfred-Kowalke-Straße 17, 10315 Berlin, Germany; 2 Department of Cardiovascular Surgery, Heart Center Brandenburg, University Hospital Brandenburg Medical School, 16321 Bernau, Germany; 3 Brandenburg Medical School, Faculty of Health Sciences Brandenburg, 14770 Brandenburg, Germany; 4 Clinical Center for Reproduction, Department for Small Animals and Horses, Vetmeduni Vienna, 1210 Vienna, Austria

**Keywords:** Corpus luteum, Luteal cells, Steroidogenesis, Felids, Spheroids

## Abstract

The study aimed to establish a long-term 3D cell culture model using luteinized follicular cells to investigate the functionality and life cycle of the CL in felids. A mixture of cell types from antral follicles was luteinized in vitro and cultured for up to 23 days. The method, initially applied to the domestic cat, was later extended to Persian and Clouded leopards. Antral follicles were isolated and digested with enzymes; then, the cells were subjected to culture. Experimental subsets were treated with/without 1 µg/mL cloprostenol to validate the cell culture model’s suitability for functional studies. In domestic cat samples, microscopic evaluation indicated luteinization, which was confirmed by increased progestagen concentrations and IHC staining for HSD3B and CYP11A1. The gene expression of selected steroidogenic factors (HSD3B1, STAR, CYP11A1) and hormone receptors (LHCGR, PTGFR, PRLR) significantly increased, while CYP17A1 expression decreased. Cloprostenol treatment resulted in reduction of steroidogenic activity, proving its suitability for functional studies. Persian and Clouded leopards’ cell cultures exhibited similar patterns in progestagen secretion and gene expression, compared to domestic cats. This model, with its defined luteinization, as well as high and stable progestagen production, allows future investigation of factors regulating CL life cycle and function.

**Supplementary Information:**

The online version contains supplementary material available at 10.1007/s00441-024-03937-z.

## Introduction

The corpus luteum (CL) is a usually transient endocrine gland located on the mammalian ovary. The gland produces progesterone, which is the main hormone that prepares the uterus for embryo implantation and helps maintain pregnancy. In some species, other progestagen are produced (Brown 2018; Conley et al. 2021). The CL develops from an antral follicle after ovulation. During luteinization and CL formation, granulosa cells (GCs) and theca cells (TCs) of the follicle differentiate into large luteal cells (LLCs) and small luteal cells (SLCs), respectively. In some species, SLCs can also differentiate into LLCs (Alila and Hansel 1984; Arikan et al. 2009). The steroidogenic SLCs and LLCs are responsible for progestagen (P) production, where the steroidogenic activity of LLCs seems to be higher than that of SLCs in different species (Hryciuk et al. 2019; Nelson et al. 1992; Ursely and Leymarie 1979). The nonsteroidogenic cells of the CL, e.g., pericytes, fibroblasts, endothelial cells, and immune cells (macrophages, lymphocytes, and neutrophils) play an important role in supporting steroidogenesis and CL regression (Fridén et al. 2000; Pate and Landis Keyes 2001; Stocco et al. 2007; Walusimbi and Pate 2013).

The domestic cat is a seasonal polyestrous breeder exhibiting induced/ reflex (Concannon et al. 1980) or spontaneous ovulation (Binder et al. 2019). Formed after ovulation, corpora lutea (CLs) enter either a pregnant or pseudopregnant luteal phase which last ± 65 days or ± 40 days, respectively (Tsutsui and Stabenfeldt 1993). Throughout their lifespan, the CL of pregnancy or pseudopregnancy undergoes similar developmental stages, as determined previously based on histological classification and steroidogenic capacities (Amelkina et al. 2015a; Zschockelt et al. 2014). After (pseudo)pregnancy, CLs of domestic cats regress to corpora albicans. Like domestic cats, Clouded leopards are seasonal breeders, usually exhibiting induced ovulation, though spontaneous ovulation can also occur (Brown et al. 1995). During pregnancy, fecal P metabolite concentrations remain elevated for approximately 89 days, or for 47 days during a pseudopregnant luteal phase (Brown et al. 1995). In contrast, knowledge about the reproductive biology of the Persian leopard is limited. It is also described as a seasonal breeder (Farhadinia et al. 2009). Different reproduction strategies and CL lifecycles, in comparison with that of the domestic cat and Clouded leopard, have been described in lynxes. In lynxes, CLs do not completely regress following the (pseudo)pregnant luteal phase. Instead, they are present on the ovary for a minimum of two years, continuously maintaining luteal function, as evidenced by stable elevated P serum levels (Painer et al. 2014). The mechanism underlying the persistence of CLs and their evolutionary significance in lynxes remains elusive. Moreover, there is a paucity of knowledge regarding CL functionality in felids in general. Previous studies, primarily conducted on domestic cats and lynxes, have provided initial insights into the characteristics of feline CL (Amelkina et al. 2015a, 2016; Braun et al. 2020; Dawson 1941, 1946; Goritz et al. 2009; Hryciuk et al. 2019; Hryciuk et al. 2021a, b; Painer et al. 2014; Zschockelt et al. 2015, 2014, 2016). A more comprehensive understanding of mechanisms and factors regulating CLs lifecycles is needed for a better general understanding of the gland and their differences between species, e.g., regression and persistence mechanisms in domestic cat and different lynx species. Such knowledge can also facilitate the successful implementation of assisted reproductive techniques, e.g., luteolysis and estrus induction.

A better understanding of the CL can be provided by functional studies performed in vitro on luteal cells, thereby obviating the requirement for animal experimentation, which is almost not feasible for felids. The currently existing luteal cell cultures for different species describe systems only including steroidogenic SLCs and/or LLCs (Arikan and Yigit 2009; Brannian and Stouffer 1991; Hryciuk et al. 2019; Kowalewski et al. 2013; Nelson et al. 1992) or hetero-cultures where the steroidogenic cells have been maintained with different types of nonsteroidogenic cells, such as immune or endothelial cells (Castro et al. 1998; Korzekwa et al. 2008; Nelson et al. 1992). For the domestic cat, the first luteal cell culture with different types of freshly dispersed luteal cells was described by Arikan et al. (2009). This group cultured the cells in a 2D system for 7 days (Arikan and Yigit 2009). In one of our previous studies, separate isolation methods and cell culture conditions for SLCs and LLCs were described (Hryciuk et al. 2019). Although our further investigations were focused on SLC functional studies (Hryciuk et al. 2021a), it appeared that SLC cultures alone were not sufficient for gaining a comprehensive understanding of CL function, particularly due to their lower steroidogenic activity in comparison with LLCs (Hryciuk et al. 2019). Our cultures with only LLCs exhibited restrictions regarding their use for a successful CL model because they showed declining viability with an increasing culture period (Hryciuk et al. 2019). Moreover, due to LLC fragility, current isolation methods for LLCs are not highly efficient (Arikan et al. 2009; Nelson et al. 1992; Weber et al. 1987; Wilkinson et al. 1976). Additional constraints stem from the small size of the CL in domestic cats and the diversity of the luteal stages, which further restrict the availability of cells for experimental purposes. The in vitro luteinization of TCs and GCs, progenitor cells of SLCs and LLCs, has been successfully performed in several species (Engelhardt et al. 1991; Meidan et al. 1990). Therefore, the luteinization of GCs in domestic cats in vitro seems to represent a way to obtain cells for the further analysis of CLs. Although the successful in vitro luteinization of GCs from domestic cats in a 3D system has been performed, apoptotic bodies were observed within the spheroid structure (Hryciuk et al. 2023), so the model does not yet seem optimal. This and the decline in the viability of LLCs cultured alone leads to the hypothesis that LLCs from the domestic cat may require support from other cells from the CL to facilitate a long-term culture (Hryciuk et al. 2023). The luteinization of a mixture of cells from antral follicles in spheroids appears to be a promising approach. To the best of our knowledge, the digestion of whole antral follicles and the use of the mixture of obtained cells for luteinization and the creation of a model for a long-term 3D culture have not yet been done, but 3D cultures with TCs and/or GCs for, e.g., 3D follicular-like model have been previously established in some species for other research questions (Hummitzsch et al. 2009; Ip et al. 2022; Jeon et al. 2021; Yadav et al. 2018).

The aim of this study was to develop and characterize a 3D cell culture model that could be used for in vitro studies on the CLs of felids. As a novelty, cells derived from antral follicles were used. After luteinization of steroidogenic cells, within population of other cells, we aimed to maintain their high steroidogenic activity. To demonstrate the practical utility of the developed system, cells were exposed to cloprostenol, a synthetic analog of prostaglandin F2α (PGF2α) known to induce luteal regression in mammals (Atli et al. 2012). Previously, we demonstrated that cloprostenol negatively affected P synthesis in luteal cell cultures of felids (Hryciuk et al. 2021a). Achieving similar effects in this new 3D culture system was expected to validate its use for functional studies. The domestic cat was used as a model species to establish the experimental protocol, which was subsequently applied to available samples from two wild felid species, the Persian leopard (*Panthera pardus tulliana*) and the Clouded leopard (*Neofelis nebulosi*). However, the results for the leopards should be considered case reports (*n* = 1).

## Materials and methods

This study was approved by the Internal Committee for Ethics and Animal Welfare of the Leibniz Institute for Zoo and Wildlife Research (IZW) (2017–02-02). All chemicals used were purchased from Merck KGaA, Darmstadt, Germany, unless otherwise stated. Samples from domestic cats, routinely ovariohysterectomized for population management reasons between May and July, were obtained from either Tierheim Berlin, Germany, or the Clinical Center for Reproduction, Department for Small Animals and Horses, Vetmeduni Vienna, Austria. The ovaries were transported to the Leibniz-IZW in a thermo-box with cold packs within 24 h after surgery. During the transport, each pair of ovaries was kept separately in a falcon tube with Dulbecco’s phosphate-buffered saline (DPBS) containing 3 g/L bovine serum albumin and 10 mL/L antibiotic antimycotic solution. The ovariectomies were performed for population management reasons and were not related to the purpose of this experiment. Only ovaries with visible antral follicles were used for the experiments.

The Persian and Clouded leopard samples were obtained under the Felid Gametes Rescue Project and were transported to the IZW under the same conditions as the samples from the domestic cats. The Persian leopard was ovariectomized in November 2022 for health reasons at Cologne Zoological Garden. Samples from the Clouded leopard were obtained in January 2023 after the death of a female at Tierpark Berlin.

### Cell isolation

The antral follicles (around 2–3 mm) were isolated by excising them from the ovary. Subsequently, the follicles were transferred to a new petri dish and cut into small pieces. The pieces of the follicles and the cells released from them were then collected in a 15 mL-tube and subjected to enzymatic digestion in an isolation medium (HAM’s F12 [PAN-Biotech GmbH, Aidenbach, Germany] and Minimal Essential Medium [MEM] Eagle with HEPES modification 1:1, supplemented with 1% FBS and Antibiotic–Antimycotic 1X) supplemented with collagenase (class I and II) and DNAse at final concentrations of 0.1% and 0.005% [w/v], respectively. The tissue was digested for 55 min at 37 °C, with intermittent mixing of the tube content during the digestion process. After digestion, the cell suspension was filtered through a 20 μm cell strainer to eliminate undigested tissue fragments. The cells were then layered onto a 40% Percoll/60% DPBS-layer and centrifuged at 1000 × g and RT for 7 min to remove erythrocytes. Cells collected from the interphase were transferred to a 1.5 mL-tube, centrifuged at 500 × g and RT for 4 min, washed in a fresh isolation medium, centrifuged again, and resuspended in the isolation medium. The cells were subsequently counted, and their viability was assessed by staining with trypan blue. The viability of the isolated cells ranged between 84 and 95% across different biological replicates for the domestic cat; for the Persian and Clouded leopards, these values were 80% and 70%, respectively. Following another centrifugation step at 500 × g and RT for 4 min, the cells were resuspended in a freezing medium (MEM Eagle supplemented with 3.04 g/L sodium bicarbonate, 20 mM HEPES, 10% FBS, and 10% DMSO) and frozen using Mr. Frosty™ (Thermo Fisher Scientific, Waltham, USA) according to the manufacturer’s instructions. The cells were stored in liquid nitrogen until needed for further cell culture experiments. The identity and ratio of different cell types were not determined before freezing or after thawing.

### Cell culture

For three biological replicates, cells from single domestic cats were used, for the fourth replicate a mixture of cells from two individuals were used. The cells were thawed by placing them in a water bath (38 ℃) for 2 min. Then, the cells were washed in a freezing medium without DMSO, centrifuged (4 min, 500 × g), and subsequently resuspended in a luteinization medium (Ham’s F12 Medium with stable glutamine and MEM Eagle with HEPES Modification 1:1, supplemented with 1% FBS, 10 mL/L Antibiotic–Antimycotic Solution, 2 µg/mL insulin, 10 µM forskolin [SERVA Electrophoresis GmbH, Heidelberg, Germany], 60 ng/mL luteinizing hormone from human pituitary [LH]) and counted. In our study, the same concentrations of insulin (2 µg/mL) and forskolin (10 µM) were utilized as previously used by Meidan et al. for bovine cell cultures (Meidan et al. 1990). For LH, we applied a concentration of 60 ng/mL for the first 12 days, mirroring the pre-ovulatory LH serum peak concentration observed in domestic cats (Wildt et al. 1981). The viability of cells after thawing was determined using trypan blue staining and ranged from 57 to 73%, depending on the specific replicate. Then, the cells were further re-suspended in the luteinization medium at a concentration of 75,000 viable cells/mL. Furthermore, the cells were seeded on ultra-low attachment (ULA) 96-well plates (Corning Incorporated, Kennebunk, USA), with 15000 viable cells per well in 200 µl luteinization medium (culturing conditions: 37 °C, 5% CO_2_). For each biological replicate, at least 16 wells with cells were prepared at the beginning of the cell culture period. On day 12, we withdrew insulin and forskolin as well as reduced the LH concentration to 10 ng/mL to reflect the oscillating LH levels (5–10 ng/mL) characteristic for pregnancy in domestic cats (Wildt et al. 1981). The medium with this modified composition is referred to as the maintenance medium. From day 14 onward, the maintenance medium was supplemented with + 1000 ng/mL cloprostenol for some spheroids. The solvent vehicle, dimethyl sulfoxide (DMSO), was present in both, the cloprostenol treatment and control group, at a concentration of 0.1% [v/v]. A medium change (150 uL) was performed on day 2, 5, 7, 9, 12, 14, 16, 19, 21, and 23. Media collected on the same day and for the same treatment group were pooled into two or three independent samples for the domestic cats and leopards, respectively, and stored at − 20 ℃ until hormone extraction. Spheroids for gene expression analysis were collected on day 0, 2, 7, 12, 14, 19, and 23. Spheroids from additional wells, not subjected for gene expression analysis, were fixed in Bouin solution and used for hematoxylin and eosin (HE) and immunohistochemistry (IHC) staining afterwards. These and spheroids collected for gene expression analysis were transferred into tubes using Cell Saver Tips (Biozym Scientific GmbH, Hessisch Oldendorf, Germany). The tubes with spheroids for RNA isolation were plunged shortly into liquid nitrogen and stored at − 80 °C until further use. Day 0 refers to the day when the cells were thawed and the cell culture and luteinization process began. For transcript abundance analysis cells from day 0 were not subjected to cell culture, after counting, samples with15000 cells were frozen. The experiment was repeated four times for the domestic cat samples (*n* = 4) and one time (*n* = 1) each for the two leopard species.

### Progestagen extraction and EIA

Steroid extraction and P determination by EIA was done as described previously (Hryciuk et al. 2019). Analysis of P was carried out with an in-house microtiter plate enzyme immunoassay using a commercial anti-P4 antibody (Sigma P1922, raised in rats to progesterone) and 4-pregnen-3,20-dione-3-CMO-peroxidase label. The cross-reactivities to other steroids were as follows: 4-pregnen-3,20-dione (progesterone), 100%; 5α-pregnan-3,20-dione (5α-DHP), 76.8%; 5α-pregnan-3ß-ol-20-one (5a), 5-pregnen-3ß-ol-20-one, 10.8%; 18.3%; < 0.1% for 5ß-pregnan-3α-ol-20-one, 20a-dihydroprogesterone, pregnandiol, 17a-hydroxyprogesterone, testosterone, estradiol, and cortisol. The intra-assay and inter-assay values for two biological samples with lower and higher concentrations were 5.2% and 10.5%, and 10.1% and 13.0%, respectively. The minimal detectable concentration for P was 130 pg/mL. The concentration of P in medium control samples was below detection rage.

For P concentration calculation, the concentration left in the medium after a partial medium change was subtracted, e.g., from P concentration measured at day 5, we subtracted 25% of value measured at day 2. Furthermore, we divided the hormone values by the number of days the medium was in a well with cells to demonstrate the concentration of hormones produced within 1 day during the specified time periods. The presented values for P were normalized with the values of day 2 within each biological replicate, and therefore, the graphs (Fig. 3) show P fold increase in comparison to day 2. This calculation was performed because of different concentration of P in different biological replicates.

### RNA isolation, reverse transcription, and real-time PCR

Total RNA was isolated with the NucleoSpin RNA Plus XS kit (Macherey–Nagel GmbH & Co. KG, Berlin, Germany) according to the manufacturer’s manual. Isolated RNA was reverse-transcribed using the PrimeScript RT Reagent Kit (TAKARA BIO INC., Kusatsu, Japan) according to the manufacturer’s manual using Oligo dT Primer (f.c.: 25 pM) and random hexamers (f.c.: 50 pM). Real time qPCR was performed as described previously (Hryciuk et al. 2019). In brief, the diluted cDNA was analyzed in a reaction volume of 10 μl. The reaction mixture comprised SsoFast EvaGreen Supermix (Bio-Rad, Hercules, USA) and primers at a final concentration of 500 nM. The PCR reactions were conducted according to the following conditions: an initial denaturation step at 95 °C (*CYP19A1*, *AR*) or 98 °C (all other genes) for 2 min, followed by 40 cycles of denaturation at 95 °C (*CYP19A1*, *AR*) or 98 °C (all other genes) for eight seconds and annealing/elongation at specific temperatures for eight seconds. Further information on the primers and their corresponding annealing/elongation temperatures can be found in Table 1.

**Table 1:** Sequence of primers used in the study

Gene	Full name	Primer sequence	Product size [bp]	Temperature [◦C]	References
*ACTB*	Actin Beta	fw: GAG CAG GAG ATG GCC ACG rv: CTC GTG GAT GCC ACA GGA	159	62	(Zschockelt et al. 2016)
*TBP*	TATA-Box Binding Protein	fw: AGA GAG CCC CGA ACC ACT G rw: TTC ACA TCA CAG CTC CCC AC	182	62.5	(Braun et al. 2012b)
*STAR*	Steroidogenic Acute Regulatory Protein	fw: AGC TTG TGG AGC ACA TGG AA rv: TGA TTC TGC AGC CAA CTC GT	116	61.5	(Zschockelt et al. 2014)
*CYP11A1*	Cytochrome P450 Family 11 Subfamily A Member 1	fw: CTT CCG GAA CCT GGG CTT rv: GCA GCG TCC ACC CTC TCT A	240	61.5	(Braun et al. 2012b)
*CYP17A1*	Cytochrome P450 Family 17 Subfamily A Member 1	fw: GAC CAG TTC ATG CCC GAA C rv: GGA CCT CCA GGT CGA ACC T	175	61.5	(Zschockelt et al. 2015)
*CYP19A1*	Cytochrome P450 Family 19 Subfamily A Member 1	fw: GATATGTGGAGAGGAAACACTCAT rv: CGAACAGCTTTCCAGAGGG	171	56.5	(Braun et al. 2012b)
*HSD3B1*	Hydroxy-Delta-5-Steroid Dehydrogenase, 3 Beta- And Steroid Delta-Isomerase 1	fw: TTG GTG GAG GAG AAG GAC C rv: CGG TGT GGA TGA TGA CTG A	181	62.5	(Zschockelt et al. 2015)
*LHCGR*	Luteinizing Hormone/Choriogonadotropin Receptor	fw: CCT GGT GTA CAT CGA GCC T rv: GGA TTC GTT ATT CAT CCC TTG	199	57.5	(Hryciuk et al. 2019)
*PTGFR*	Prostaglandin F Receptor	fw: GCT GGA GTC CAT TTC TGG TG rv: CCA CGT TGC CAT TCG AAG	104	61	(Zschockelt et al. 2016)
*AR*	Androgen Receptor	fw: GGA ACT TGA TCG TAT CAT TGC rv: CAT TTC TGG AAA GTC CAC G	176	56.5	(Amelkina et al. 2016)
*PRLR*	Prolactin Receptor	fw: GCT CAC ACT CCA GTA CGA AA rv: TCT GTC CTG GAT ATA AGC TGA	112	60.5	(Hryciuk et al. 2019)
*VEGF*	Vascular Endothelial Growth Factor A	fw: CCT TGC CTT GCT GCT CTA C rv: CCT GGA AGA TGT CCA CCA G	158	59.5	This study
*COL1A1*	Collagen Type I Alpha 1 Chain	fw: CCT AAA GGT GCT GCT GGA G rv: CCA GGA AGA CCC TGG AAT C	188	59	This study
*CASP3*	Caspase 3	fw: ACC GGC AAA CCC AAA CTC rv: CTG ACA GGC GAT GTC ATC C	102	60,5	(Amelkina et al. 2015b)

The quantification of the RT-qPCR products was carried out using Bio-Rad CFX Manager 3.1 Software. To ensure standardization, serial dilutions of gene-specific PCR-products or plasmid DNA carrying a sequence of the target gene were employed as standards. The results obtained for the target genes were normalized using a factor calculated with the assistance of qbase PLUS software from Biogazelle (Vandesompele et al. 2002) based on reference gene expressions. *ACTB*, *TBP*, *GAPDH*, *RPS7*, and *B2M* were tested as potential reference genes, and *ACTB* and *TBP* were found to be most suitable as reference genes based on analysis with qbase PLUS and were used for normalization. In order to account for potential contamination or technical artifacts, the analysis included samples for no template control (NTC) and no reverse transcriptase control (NRT).

For the domestic cat, the following genes were chosen for analysis: *HSD3B1*, *STAR*, *CYP11A1*, *CYP17A1*, *CYP19A1*, *LHCGR*, *PTGFR*, *AR*, *PRLR*, *VEGF*, *COL1A1*, and *CASP3*. For the samples from the leopard species, the following genes were analyzed: *STAR*, *LHCGR*, *PTGFR*, and *PRLR*.

### Histology/immunohistochemistry

Collected spheroids, cells pelleted after isolation or isolated CL from the domestic cat were subjected to HE and IHC staining. After fixation, spheroids and cell pellet were embedded in 1% agarose. These agarose blocks underwent the usual histological dehydration scheme before they were embedded in paraffin. The HE staining was performed according to a standard protocol. The IHC was performed as described previously in Braun et al. (Braun et al. 2012a). Details for the antibodies are presented in Table 2. The results of the HE and IHC staining were analyzed under an inverted microscope (IX81, Olympus Deutschland GmbH, Hamburg, Germany). Pictures were taken with a DP72 camera (Olympus) and the cellSens imaging software (Olympus).

**Table 2:** Description of primary and secondary antibodies used for immunohistochemistry

	Antibody	Host	Type	Dilution	Source
CYP11A1	Anti-CYP11A1 antibody (D-12) (BSS-BS-3608R-100)	rabbit	primary	1:2500	Thermo Fisher Scientific
	Anti-rabbit-POD: ImmPRESS*-VR ANTI-RABBIT IgG KIT, HRP, Horse (horseradish peroxidase) (VEC-MP-6401–15)	horse	secondary	ready to use	BIOZOL Diagnostica Vertrieb GmbH, Eching, Germany
HSD3B1	Anti-3β-HSD Antikörper (37–2) (sc-100466)	mouse	primary	1:100	Santa Cruz Biotechnology Inc., Heidelberg, Germany;
	Anti-mouse-POD: ImmPRESS™ VR REAGENTAnti-Mouse IgGVeterinary ReagentPEROXIDASE (Vec-MP-6402–15)	horse	secondary	ready to use	BIOZOL Diagnostica Vertrieb GmbH, Eching, Germany

### Statistics

This study presents the results of four biological replicates (*n* = 4) from the domestic cat. One of the biological replicates consists of cells from two cats, while the other three biological replicates consist of cells from a different individual each. For each biological replicate, we had two technical replicates (cells cultured in separate wells) at all the analyzed time points.

Statistical analysis was performed using R (R Core Team, Vienna, Austria). For each gene, the measured relative mRNA level at day 0, 2, 7, 12, 14, 19, and 23 was compared to that at day 0, day 14, and day 23 respectively. The data structure at hand, including results for two technical replicates for each of the four biological replicates at each time point, reflects a partly dependent, partly independent dataset, corresponding to a crossed and nested design (Krzywinski et al. 2014). Therefore, we refrained from using (paired) Student’s *t*-tests or their non-parametric counterparts, respectively, as well as a nested ANOVA. Instead, we performed a series of linear mixed models, comparing two time points each, using the R packages lme4 (Bates et al. 2015) and lmerTest (Kuznetsova et al. 2017). The model was designed using the relative mRNA expression level of selected gene as dependent variable, the biological replicates number as subjects, the day of measurement as fixed factor, and a random slopes model for the influence of the measurement day. The later was chosen after comparing mixed models with and without random slopes for each value of relative mRNA expression level using the ANOVA function to access goodness of fit, which implicated a non-neglectable subject dependency of the influence of the measurement day. While the majority (68%) of data was normally distributed according to Shapiro–Wilk tests, some comparisons were made with non-normally distributed data. Similarly, > 80% of comparisons were between pairings with variance homogeneity, as tested by Levene test. Linear mixed modeling has been shown to be robust against violations of distribution assumptions (Schielzeth et al. 2020) though. In the interest of not further complicating the analysis, we deemed these terms acceptable to perform the analysis as stated. This described procedure resulted in 7 *P* values for each of the 3 chosen reference points (day 0, day 14, day 23). To account for alpha-error accumulation due to multiple testing, *P* values of the comparisons respectively were corrected using Holm Bonferroni method (Abdi 2007).

The same approach was employed to quantify changes in P concentration during the cell culture period. We used values corresponding to daily P production within specific time periods. This was necessary because the medium was changed every 2 or 3 days (medium change on day 2, 5, 7, 9, 12, 14, 16, 19, 21, and last collection on day 23).

In the experiment testing the cloprostenol influence on transcript abundance levels or P production, the statistical method was similar to the abovedescribed method, but linear models were built for each day.

The results are shown as a boxplots with medians. The groups that are statistically different are described with bars indicating the significance level (e.g., *P < 0.05; **P < 0.01; ***P < 0.001). For the Persian and Clouded leopard samples, we present results from single biological replicate for each species (*n* = 1), with three technical replicates for each selected time point. Statistical analysis was not performed for the results from the Persian and Clouded leopards because only a single biological replicate of each was available.

## Results

### Characterization of cells during the luteinization/maintenance culture period

#### Spheroid formation and morphology

**Fig. 1:**
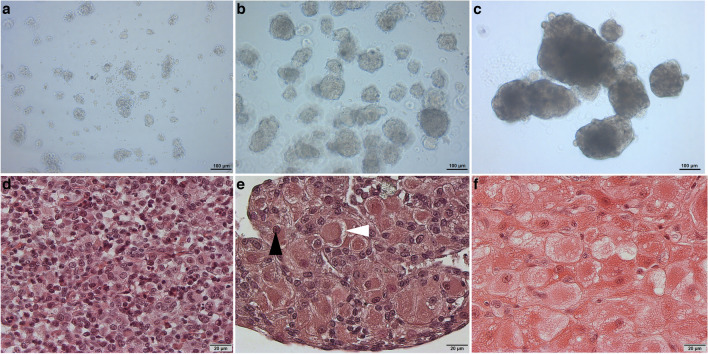
Photomicrographs of cells from antral follicle of domestic cats during in vitro luteinization and maintenance: day 1 (**a**); day 5 (**b**); day 23 (**c**). Hematoxylin- and eosin-stained histology sections from domestic cat: mixture of isolated cells from antral follicles (**d**), spheroid with luteinized cells of antral follicles after 14 days in culture (**e**), CL of domestic cat from development/maintenance stage (**f**) for comparison purpose. The black arrowhead points to the vesicular nucleus; the white arrowhead points to lipid droplets

Upon transferring the cells into a cell culture well, for all the species it was observed that they rapidly initiated the process of aggregation, resulting in the formation of small spheroids (Fig. 1a, Supplementary file 1). These small spheroids subsequently merged together, leading to the formation of either a single or multiple spheroids at the final stage (Fig. 1b–c, Supplementary file 1).

The HE staining was performed on freshly isolated cells from the antral follicles of domestic cats (Fig. 1d), spheroids cultured for 14 days (Fig. 1e), and a CL from the development/maintenance stage for structural comparison (Fig. 1f). The freshly isolated cell mixture exhibited small cells (Fig. 1d). Within the spheroids (Fig. 1e), a heterogeneous population of cells with varying sizes and morphologies was observed. Some cells displayed smaller sizes and seemed to have a nucleus-to-cytoplasmic-area ratio similar to the freshly isolated cells. In contrast, the other cells within the spheroids were notably larger and had greater cytoplasm to nucleus ratio. Furthermore, these larger cells exhibited numerous small vacuoles or lipid droplets and had vesicular nucleus. The larger cells forming the spheroids were of comparable sizes with those observed in the CL from the maintenance/developmental stage (Fig. 1f). Furthermore, cross-sections showed a higher presence of small cells within the spheroids when compared to CL.

#### Protein expression analysis of CYP11A1 and HSD3B by immunohistology

**Fig. 2:**
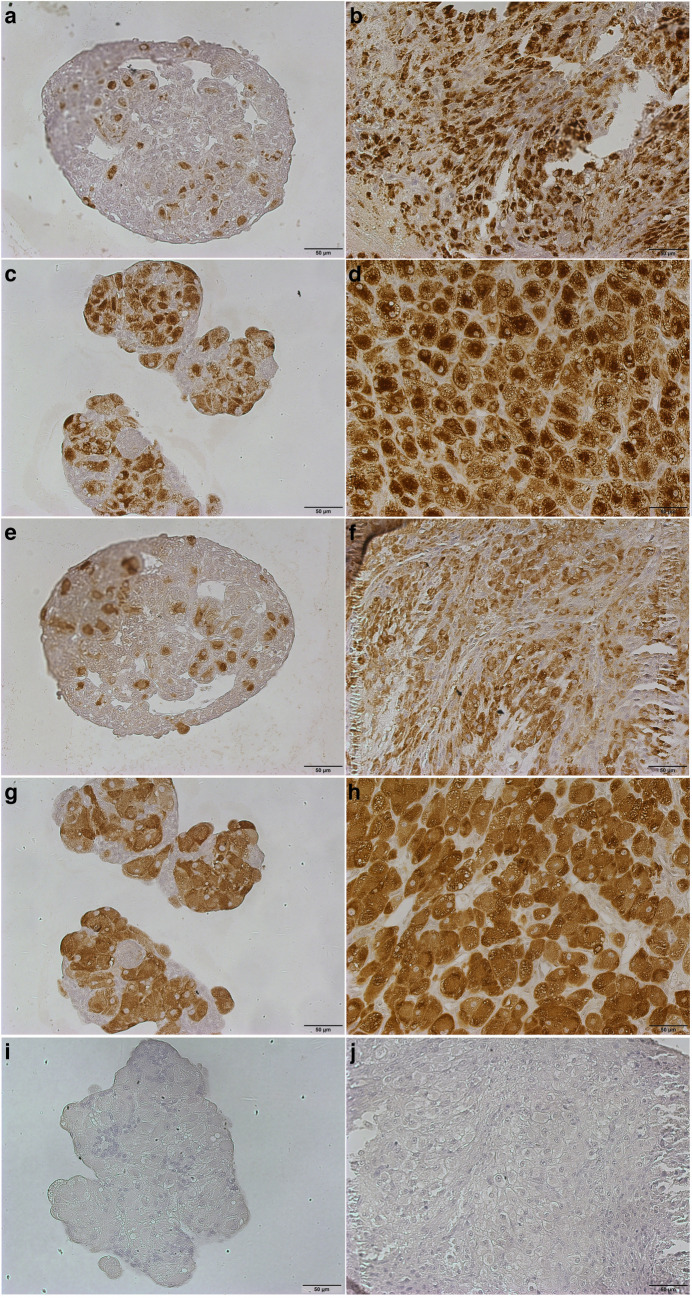
Photomicrographs illustrating immunohistochemical staining for CYP11A1 (**a**–**d**) and HSD3B (**e**–**h**) in domestic cat cells/tissues: spheroid with luteinized follicle cells at day 7 (**a** and **e**); spheroid with luteinized follicle cells at day 23 (**c** and **g**); corpus luteum in formation stage (**b** and **f**); corpus luteum in development/maintenance stage (**d** and **h**); negative control without 1^st^ antibody and anti-mouse-POD as second antibody (spheroid (**i**) and CL (**j**))

IHC staining was performed to assess the protein expression of CYP11A1 and HSD3B, markers for steroidogenic cells, in spheroids after 7 and 23 days of culture, as well as in CLs at the corresponding formation (for day 7) and development/maintenance (for day 23) stages (Fig. 2). The results show that both the spheroids and felid CL contain cells that stain positively for CYP11A1 (Fig. 2a–d), and HSD3B (Fig. 2e–h). Furthermore, some cells did not exhibit staining for CYP11A1 and HSD3B in both the spheroids and CL tissue. Control staining is presented in Fig. 2i and j.

#### Progestagen concentration

**Fig. 3:**
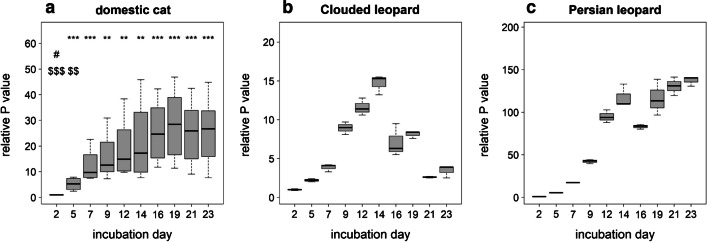
Relative *P* values in medium produced per day in: domestic cat (**a**), Clouded leopard (**b**), and Persian leopard (**c**). The relative values based on normalization of per day concentrations with P concentration for day 2. *, #, and $ indicate significance level (e.g., *P < 0.05; **P < 0.01; ***P < 0.001) between day 2 (*), 14 (#), and 23 ($) against all other time periods, respectively. Statistical analysis was not performed for the Clouded and Persian leopards (*n* = 1). For the leopard species, the box plots were built based on values for technical replicates

In the media of cultured cells from the domestic cats, P concentrations were measured throughout the entire duration of the experiments, as shown in Fig. 3a. The lowest relative per-day value of P was measured at day 2 and was significantly increased at all further measured days. No significant changes between the different time points were detectable for day 7 to day 23 in comparison with day 23.

In the samples from the Persian and Clouded leopards, EIA measurements were performed for P too. For the samples from the Clouded leopard, relative P value was increasing until day 14 (*n* = 1, no statistics performed) and then presented a decreasing pattern, whereby the last P value was comparable to the one calculated for day in 7 (Fig. 3b). The calculated P values of the samples from the Persian leopard appeared to increase throughout the entire culture period (*n* = 1, no statistics performed) (Fig. 3c).

#### Transcript abundance of selected gene

**Fig. 4:**
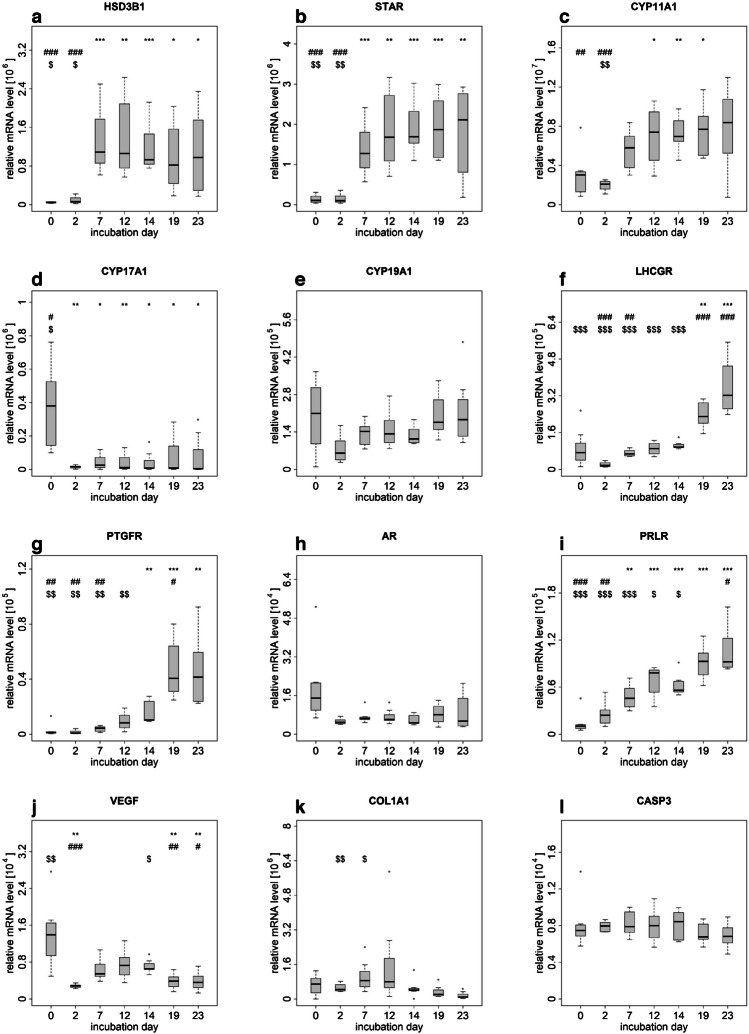
Relative mRNA expression levels of selected genes in cells of antral follicles from domestic cats before (day 0) and during culture period (day 2, 7, 12, 14, 19, 23). *, #, and $ indicate significance level (e.g., *P < 0.05; **P < 0.01; ***P < 0.001) between day 0 (*), 14 (#), and 23 ($) against all other values, respectively

All the genes investigated in this study were detected in the analyzed samples, and their expression exhibited gene-specific patterns (Fig. 4). For the domestic cat, mRNA abundance was analyzed on day 0, 2, 7, 12, 14, 19, and 23. Day 0 refers to the cells after thawing. Notably, statistical analysis did not reveal significant differences during the culture period for gene expression of *CYP19A1*, *AR*, and *CASP3* (Fig. 4e, h, and l). The expression values for *HSD3B1* did significantly change, with the values for day 0 and 2 being significantly lower compared to the values measured on day 14 or 23 and the values on day 7, 12, 14, 19, and 23 being significantly higher than the value on day 0 (Fig. 4a). The same gene expression pattern, with even partly higher significance levels on some days, was detected for *STAR* (Fig. 4b). Furthermore, for *CYP11A1*, a statistically significant increase in abundance was measured between day 0 and day 12, 14, and 19, whereas the level at day 14 was significantly higher compared to day 0 and 2 as well as at day 23 compared to day 2 (Fig. 4c). For *CYP17A1*, the abundance measured at day 0 was significantly higher than on any other day of the culture period (Fig. 4d). Additionally, a significant increase in abundance was detected for *LHCGR, PTGFR*, and *PRLR* across the different time points, as illustrated in Figs. 4f, 4g, and 4i. Some variations in abundance were also detected for *VEGF* and *COL1A1* but with different patterns (Fig. 4j and k).

**Fig. 5:**
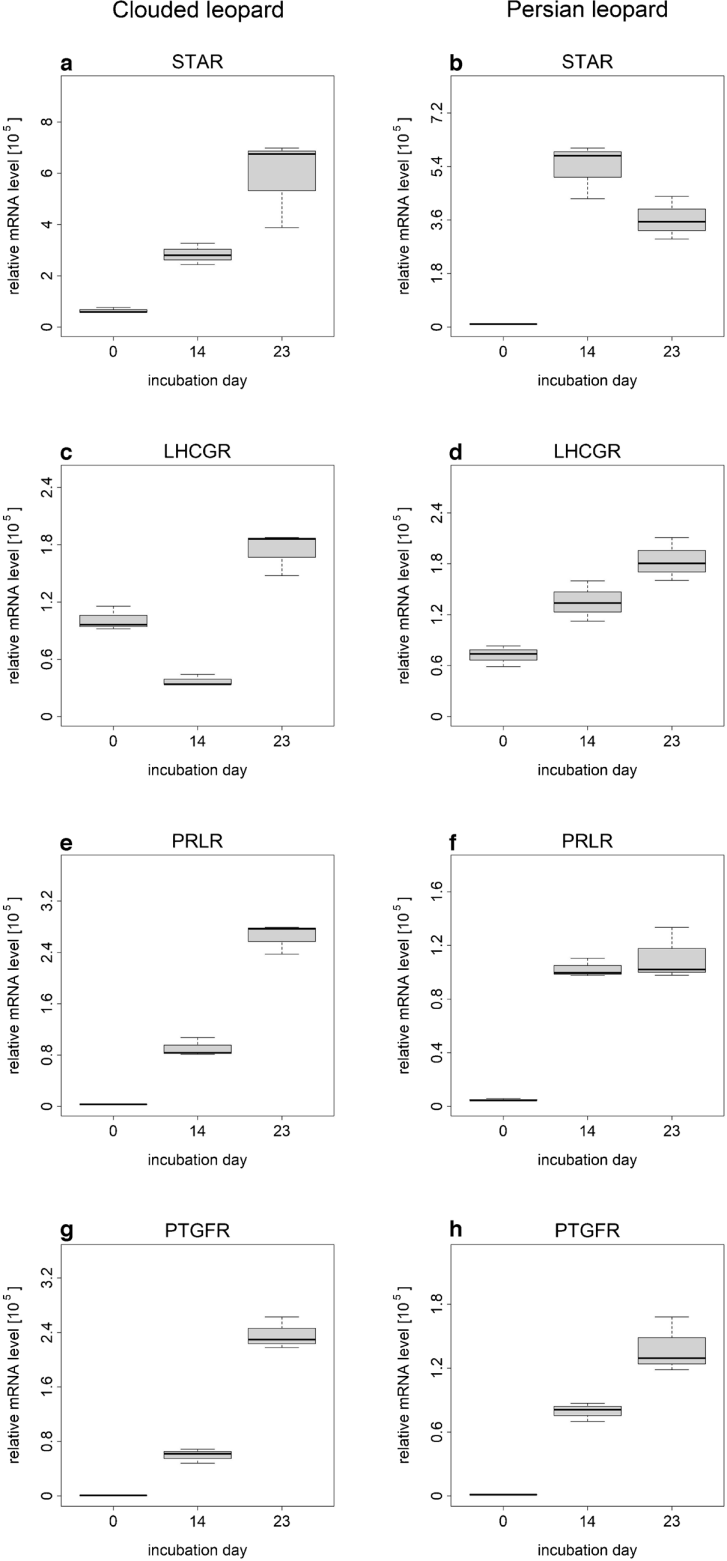
Relative expression levels of selected genes in cells of antral follicles from Persian leopard and Clouded leopard before (day 0) and during culture period (day 14 and 16). Statistical analysis was not performed (*n* = 1). Box plots were built based on values for technical replicates

The cells from the two leopard species were analyzed for the mRNA abundance of *STAR*, *LHCGR*, *PTGFR*, and *PRLR* on day 0, 14, and 23 of the culture period (Fig. 5). The transcript abundance pattern during culture period is depicted in Fig. 5. For all tested genes, a higher expression was detected on day 23 compared to day 0. No statistical analysis was performed (*n* = 1).

### Functional test of the spheroid model: treatment of cells with cloprostenol

#### Progestagen concentration

**Fig. 6:**
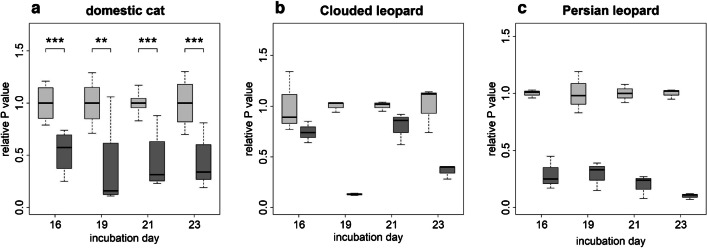
Influence of 1 µg/mL cloprostenol on relative P production by luteinized cells from antral follicle of domestic cat (**a**), Clouded leopard (**b**), Persian leopard (**c**). Light grey boxes indicate control group; dark grey boxes indicate cloprostenol treatment group. * indicates significance level; **P < 0.01; ***P < 0.001. Statistical analysis was not performed for Clouded leopard and Persian leopard (*n* = 1). The presented values, for control and treatment group, were normalized with the average of the technical replicates of control group from the same culture day

Cloprostenol reduced the P production of the cultured luteinized cells from the domestic cat (Fig. 6a) and the two leopard species (Fig. 6b and c). In the domestic cat, a statistically significant decrease in P secretion was determined at all treatment periods compared to their respective controls (Fig. 6a). No statistical analysis was performed for samples of Persian and Clouded leopards (*n* = 1).

#### Transcript abundance of selected gene

**Fig. 7:**
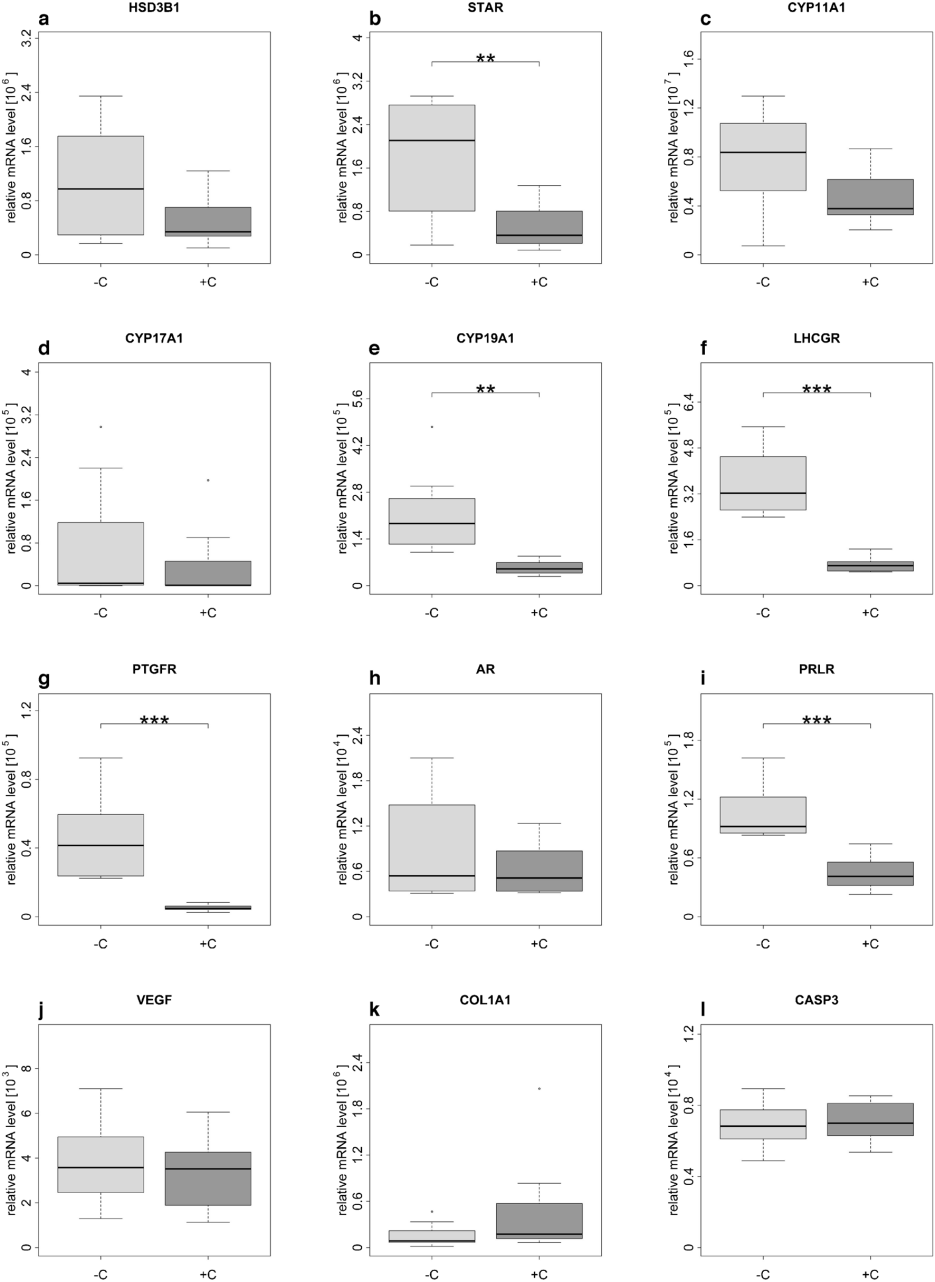
Influence of + / − 1 µg/mL cloprostenol on gene expression of selected genes on day 23 of culture period in domestic cat. Cloprostenol treatment started on day 14. Light grey boxes indicate on control group; dark grey boxes indicate cloprostenol treatment group. * indicates significance level; **P < 0.01; ***P < 0.001

**Fig. 8:**
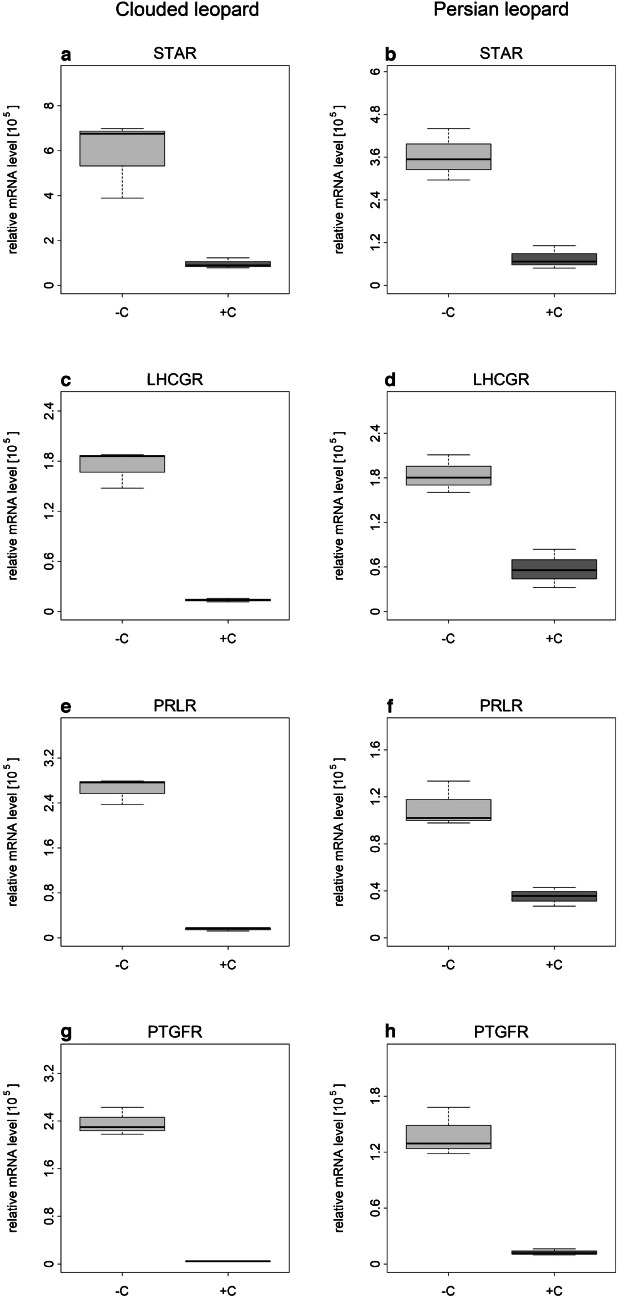
Influence of + / − 1 µg/mL cloprostenol on gene expression of selected genes on day 23 of culture period in Clouded and Persian leopards. Cloprostenol treatment started on day 14. Light grey boxes indicate control group; dark grey boxes indicate cloprostenol treatment group. Statistical analysis was not performed (*n* = 1). Box plots were built based on values for technical replicates

Gene expression analysis was conducted on cells treated with cloprostenol for 9 days (between day 14 and 23 of the culture period, harvested on day 23) and compared with the untreated cells. In the domestic cat, the treatment caused a statistically significant decrease in the mRNA abundance for *STAR* (Fig. 7b), *CYP19A1* (Fig. 7e), *LHCGR* (Fig. 7f), *PTGFR* (Fig. 7g), and PRLR (Fig. 7i) compared to the control group. In contrast, for the remaining genes examined (*HSD3B1*, Fig. 7a; *CYP11A1*, Fig. 7c; *CYP17A1*, Fig. 7d; AR, Fig. 7h; *VEGF*, Fig. 7j; *COL1A1*, Fig. 7k; *CASP3*, Fig. 7l), no significant changes were observed. Similarly, the treatment with cloprostenol resulted in a lower mRNA abundance of selected genes (Fig. 8) in samples of Clouded and Persian leopards (*n* = 1, no statistics performed).

## Discussion

This work developed a novel 3D cell culture model to study CL function of felids based on the luteinization of cells derived from antral follicles. The current model is an improvement of our previous model with only GC luteinized in 3D culture (Hryciuk et al. 2023) because it allows to maintain sustainable steroidogenic activity and seems to prevent luteal cell apoptosis.

In our cell culture system, during the first 12 days of culture, we used three factors to stimulate luteinization: insulin, forskolin, and LH. This approach builds on previous work by Meidan et al. (1990), who successfully used insulin and forskolin for the luteinization of bovine GCs, and our previous work on luteinization of GC of domestic cat (Hryciuk et al. 2023). After 12 days, we changed to a medium without forskolin and insulin and reduced the LH concentration. This change in medium composition did not affect the steroidogenic activity of luteinized follicle cells, as indicated by calculated relative P values (day 12 vs. day 14) and the transcript abundance of genes involved in steroidogenesis (day 12 vs. day 14).

In our study, we did not induce spheroid formation via centrifugation, as described in another study (Jeon et al. 2021). In contrast, we used ULA plates, the coating of which causes cells to not attach to the plate but instead aggregate with each other (Langhans 2018). This is additionally facilitated by the “u” shape of the well. Furthermore, we did not manipulate the cell distribution within the spheroids, as also described in the other study (Jeon et al. 2021), where the GCs were cultured first to create the core of the spheroids before adding the TCs to create the outer layer of the spheroids. In the CL of the domestic cat, non-steroidogenic cells are dispersed throughout the CL tissue, and their ratio seem to change during CL lifespan (Amelkina et al. 2015a). Our spheroids with luteinized cells of antral follicles exhibited a mixture of different cell types. When comparing the HE-stained sections of CLs from the development/maintenance stage of a pseudopregnant cat (Fig. 1f) with our spheroids containing luteinized cells from antral follicles (Fig. 1e), there appeared to be a greater presence of small (presumably non-steroidogenic) cells in the spheroids. The reason for this could be due to the isolation process, where we likely isolated, along with a mixture of follicle cells, some follicle-surrounding cells that would not naturally be part of the developing CL. However, since these cells were present in the initial culture mixture, they were incorporated into the spheroid. Nevertheless, the steroidogenic luteal/luteinized cells seemed to be comparable in size. The characteristic layer of small lipid droplets (type I vacuolation) at the cell periphery of the large cells in spheroids of day 23 as well as their large, round, and vesicular nucleolus (Fig. 1e) aligns with the description of luteal steroidogenic cells in CLs during the development/maintenance phase of the domestic cat, as reported by Amelkina et al. (2015a) and shown in Fig. 1f. The detection of steroidogenic cell markers CYP11A1 (Rosal et al. 2022) and HSD3B (Yoshioka et al. 2013) in these large cells in spheroids (Fig. 2c and g) confirmed their steroidogenic property.

As described in the introduction, we observed apoptotic bodies scattered across spheroids in which only GCs were used for luteinization (Hryciuk et al. 2023). We suggested that the luteinization of a cell mixture could prevent this and could lead to a generally improved luteinization and steroid production. We therefore decided to study the luteinization of an antral cell follicle mixture in more detail. First, we did a preliminary experiment where the P secretion of spheroids generated by luteinized mixed cell populations derived from antral follicles was compared to P secretion of spheroids generated by luteinization of GC or TC only. The spheroids of the luteinized follicular cell mixture produced considerably more P than luteinized GCs or TCs (Supplementary file 2). Furthermore, in the present study, we did not observe apoptotic bodies scattered across the spheroids with luteinized follicle cells. Presumably, the improvements of this new cell culture system are due to the digestion of whole antral follicles leading to the presences of a variety of cell types so that (luteinized) GC and TC could be supported by other cells types, as described in other species (Nelson et al. 1992). Another explanation is that the isolation method used in our previous experiment (Hryciuk et al. 2023) resulted in the collection of a different subpopulation of GC. A further reason for the increased P secretion in our new culture system could be that only cells from large antral follicles were used, whereas in our previous and preliminary study, GCs came from follicles of various sizes.

In our study, we used a mixture of different cell types from antral follicles of similar sizes, isolated between May and July during the breeding season of domestic cats. After isolation of the cells, at least GCs, TCs, endothelial cells, and fibroblasts were detected based on known marker genes: GC, *CYP19A1* (Hatzirodos et al. 2015); TC, *CYP17A1* (Hatzirodos et al. 2015); fibroblast cells, *COL1A1* (Muhl et al. 2020); and endothelial cells, *VEGF* (Murohara et al. 1999). Looking at the gene expression of these markers in the individual samples, we observed substantial variation between samples (Supplementary file 3). The exact ratio of all potentially present cell types, could, however, not be determined. One potential reason for variation in cell composition is the different number of GC that exists in various follicles and individuals (Munakata et al. 2018). The variation of cell types in our sample set seems to have no influence on the general P expression profile during luteinization of our experiment (Fig. 3a) although the individual P levels of the single experiments may differ. Large variations among serum P concentrations were described in different queens before (Siemieniuch et al. 2012). Sorting the different cell types after isolation and using defined cell type ratios based on this seems almost impossible to us. This is due to the variety of cell types, the relatively small number of cells that can be obtained from cat follicles, and the lack of suitable surface markers characterized for all the different cell types in the domestic cat. Such determination would also lead to an additional loss of the limited material. We are convinced that the disadvantage of not knowing the exact composition of the starting material and the spheroids is outweighed by the advantages offered by our cell culture model approach (successful luteinization of steroidogenic cells, high and stable P production afterwards, no apoptotic bodies in the spheroids, system that is usable for functional studies). We are aware that the dynamic composition of the CL, especially regarding angiogenesis or invasion of immune cells cannot be reconstructed by this in vitro model. However, reconstructing the exact composition of cell types in our spheroids as found in CL was not the aim of this study.

The successful luteinization in our system was confirmed by changes in hormone secretion, the morphology of the cells, and mRNA and protein expression. In general, the strong increase in P concentration at the beginning followed by a constantly high P concentration until the end of the culture period (day 23) in our experiment nearly resembles the pattern of serum P concentrations in the domestic cat after mating. In cats, P level shows a sharp increase in the first 10 days, followed by more or less constant high values for at least the next 15 days (Verhage et al. 1976). We speculate that the P increase in our culture system is at least partially related to the hypertrophy of cells. But hyperplasia could be another explanation as it was documented in, e.g., cow (Yoshioka et al. 2013). The P profile of spheroids from Persian leopard cells roughly fits to the fecal P profile during early pseudopregnancy or pregnancy of Arabian leopard, where a strong increase in P concentration was measured for few days after ovulation (de Haas van Dorsser et al. 2007). This comparison between two leopard subspecies was made because hormonal reproduction data are not available for Persian leopard. For the spheroids of the Clouded leopard follicle cell mixture, the P profile does not fully align with the fecal P profile in pregnant or pseudopregnant females. In those cases, the P concentration increased during the first 20 days of the (pseudo)pregnant luteal phase, followed by a period where P concentrations remained relatively stable before decreasing at the end of (pseudo)pregnancy (Brown et al. 1995). However, in our cell culture system, P started to decrease already from day 14 on. Despite this decrease, microscopic observations indicated that the Clouded leopard spheroids remained in good condition (Supplementary file 1). As there is only one biological replicate, it is not possible to determine whether this is a species-specific difference or whether it is due to this single sample.

In the created cell culture system, we measured a significant increase in *HSD3B1*, *STAR*, and *CYP11A1.* The protein products of all these genes are involved in de novo P synthesis in CLs during different steps of steroidogenesis. So, their increased gene expression points to a rapid amplification of the P synthesis system. Based on the presented results for P secretion, the gene expression of *STAR* and *HSD3B1* and the detected protein expression of CYP11A1 and HSD3B, we suggest that our cell culture system with luteinized follicle cells of antral follicles is a good model to study CL of felids. This is supported by the stable mRNA abundance and P concentration from days 7 and 5, respectively, through to the end of the culture period on day 23. In this study, we determined a decrease in the abundance of *CYP17A1* at the beginning of luteinization and then consistently lower levels in the luteinized cells in comparison with the isolated cells. In general, the downregulation of the CYP17A1 enzyme hints at a androgen-producing to progesterone-producing transformation of luteinized cells (Magoffin 2005). In some species, the expression of *CYP17A1* after luteinization is silenced (Magoffin 2005); however, this is not the case in the domestic cat. The constant expression level in the luteinized follicle cells seems to be consistent with the expression in the CL tissue of the domestic cat, where *CYP17A1* expression was not influenced by the luteal stage (Zschockelt et al. 2014). Also, the mRNA abundance of hormone receptors in the spheroids from the present study with luteinized follicle cells seems to match their expression in the CLs of the domestic cat (Swanson et al. 1995). During the culture period, the *LHCGR* abundance increased in the spheroids of the present study as well as during the culture of in vitro luteinized GCs of the domestic cat in our previous study (Hryciuk et al. 2023) and in developing CLs of domestic cats in a study from another group (Swanson et al. 1995). In the CL of other species, *LHCGR* expression increases during the luteal phase as well (Convissar et al. 2019). In the present study, *PTGFR* mRNA expression increased in the spheroids during the culture; the same was also reported between the formation and development/maintenance stages in the CLs of domestic cats (Zschockelt et al. 2016). Similarly, an increase during the life cycle of the CL has been described in macaques and cows (Kim et al. 2015; Shirasuna et al. 2012). In the present study, when analyzing the expression of a marker for fibroblasts, *COL1A1*, there was no statistical difference between the gene expression level at the beginning and the end of the culture period, which indicates that our cultures with diverse cell populations were not overgrown with fibroblasts. For *VEGF* a decrease of expression over time (Fig. 4j) was observed what could hint to a loss of endothelial cells. In general, the gene expression pattern measured for the domestic cat samples was similar to the gene expression patterns measured in the Clouded and Persian leopards.

The functional studies presented here were performed to verify whether spheroids with luteinized follicle cells can be used as a model to study CL activity of felids. PGF2α is known as a luteolytic agent that cause functional luteal regression in mammals (Atli et al. 2012). Several studies have also described its influence on P serum concentration and termination of pregnancy in domestic cats (García Mitacek et al. 2012; Nachreiner and Marple 1974; Verstegen et al. 1993; Wildt et al. 1979). PGF2α/cloprostenol was not effective to induce abortion in queens up to 21–22 day of pregnancy (García Mitacek et al. 2012; Wildt et al. 1979) and P serum concentration was not influenced by injections with PGF2α at days 4–5 and 12–13 (Wildt et al. 1979). However, a rapid decrease in P concentration and termination of pregnancy was possible from day 33 on (Verstegen et al. 1993). We speculate that the decrease in P secretion caused by cloprostenol in spheroids in our cell culture system, observed as early as 14 days after the start of luteinization, was due to the absence of luteotrophic factors from outside the CL that likely prevent abortion and/or P decline in the in vivo experiments described above. Furthermore, the influence of cloprostenol was previously investigated on SLCs from domestic cats and several wild felid species in a 2D culture, where its administration resulted in decreased P secretion too (Hryciuk et al. 2021a). In the present study, we tested if the above-described functional effect of PGF2α/cloprostenol could also be observed and thus help to validate the suitability of the spheroids as a model for studying the CL of felids. The concentration of cloprostenol used in in vitro experiments was discussed before (Hryciuk et al. 2021a), and for this study, we aimed to use the same concentration. Cloprostenol caused a decrease in P secretion, an effect similar to that observed in cultured SLCs of the domestic cat (Hryciuk et al. 2021a). In contrast to this previous study, the negative influence of cloprostenol on P production was continuously present over the test period of 9 days, while, in the previous study with SLCs, a decrease was present only on 1 day of the 2-day treatment period (Hryciuk et al. 2021a). In regard to gene expression, similar results for cloprostenol treatment were found between SLCs in a 2D culture (Hryciuk et al. 2021a) and the spheroids of luteinized cells from antral follicles in a 3D culture performed in the present study. A downregulation in gene expression for *LHCGR*, *PTGFR*, and *PRLR* and the lack of an influence of cloprostenol on *HSD3B1*, *CYP11A1*, *AR*, and *CASP3* was detected in both, the previous (Hryciuk et al. 2021a) and the present studies. However, in the present study, we determined a decrease in the expression of *STAR* as well. STAR is responsible for the transport of cholesterol from the outer to the inner mitochondrial membrane, where it serves as a substrate for the side-chain-cleavage enzyme (P450scc/CYP11A1) (Kowalewski et al. 2009). SLCs, due to their characteristics, generally produce less P than LLCs (Hryciuk et al. 2019). We therefore speculate that the expression of *STAR* and the influence of cloprostenol on its expression are higher in the present study with spheroids containing SLC- and LLC-like cells. Furthermore, the more pronounced response of the cells to cloprostenol in the present study may be caused by a potentially higher PGF2α-receptor content of the LLC-like cells compared to SLCs, as was observed in ovine cells for LLCs and SLCs (Braden et al. 1988). LLCs could mediate the luteolytic response of SLCs. One study indicated that interactions between different types of cells are needed for luteolytic PGF2α action and the most appropriate in vitro system should include LLCs (Korzekwa et al. 2008). The results of this study indicate that cloprostenol/PGF2α alone cause functional regression; and further changes related to the disappearance of the CL have to be mediated by other components (e.g., migrated immune cells) with or without an interplay with cloprostenol/PGF2α. For different species, macrophages and T lymphocytes are proposed to play a central role in structural and functional CL regression (Abdulrahman and Fair 2019; Bauer et al. 2001; Niswender et al. 2000; Pate and Landis Keyes 2001; Walusimbi and Pate 2013).

Our study demonstrates the successful luteinization and formation of spheroids from different cell types originating from the antral follicles of domestic cats, Persian leopard, and Clouded leopard. Although the composition of the spheroids is not fully characterized in terms of cell types and their ratios, the presented model improves existing cell culture systems of steroidogenic cells of felids by maintaining high steroidogenic activity over an extended period in a fashion which is expected of luteal cells from a felid CL. Furthermore, the spheroids exhibit sensitivity to PGF2α (closprostenol) in a manner that is comparable to SLC of felids in vitro, providing its utility as a model for CL in functional studies.

## Supplementary Information

Below is the link to the electronic supplementary material.

Supplementary file1 Photomicrographs of spheroids from the domestic cat, Clouded leopard, and Persian leopard after 9 and 23 days in culture, respectively. (PNG 29.0 KB)

Supplementary file2 Preliminary experiment - comparison of steroidogenic activity of spheroids composed of different cell types. (DOCX 25.2 KB)

Supplementary file3 Relative mRNA expression levels of marker genes for different biological replicates at day 0 and 23. (PNG 47.1 KB)

## Data Availability

The data presented in this study are available on request from the corresponding author.
