# Supplementary figures and images for: Development of a 3D in vitro model to study corpus luteum of felids based on luteinized cells from antral follicles
Source: Cell Tissue Res. 2024 Dec 19;399(2):211–29. doi: 10.1007/s00441-024-03937-z (PMC11787223; doi:10.1007/s00441-024-03937-z)

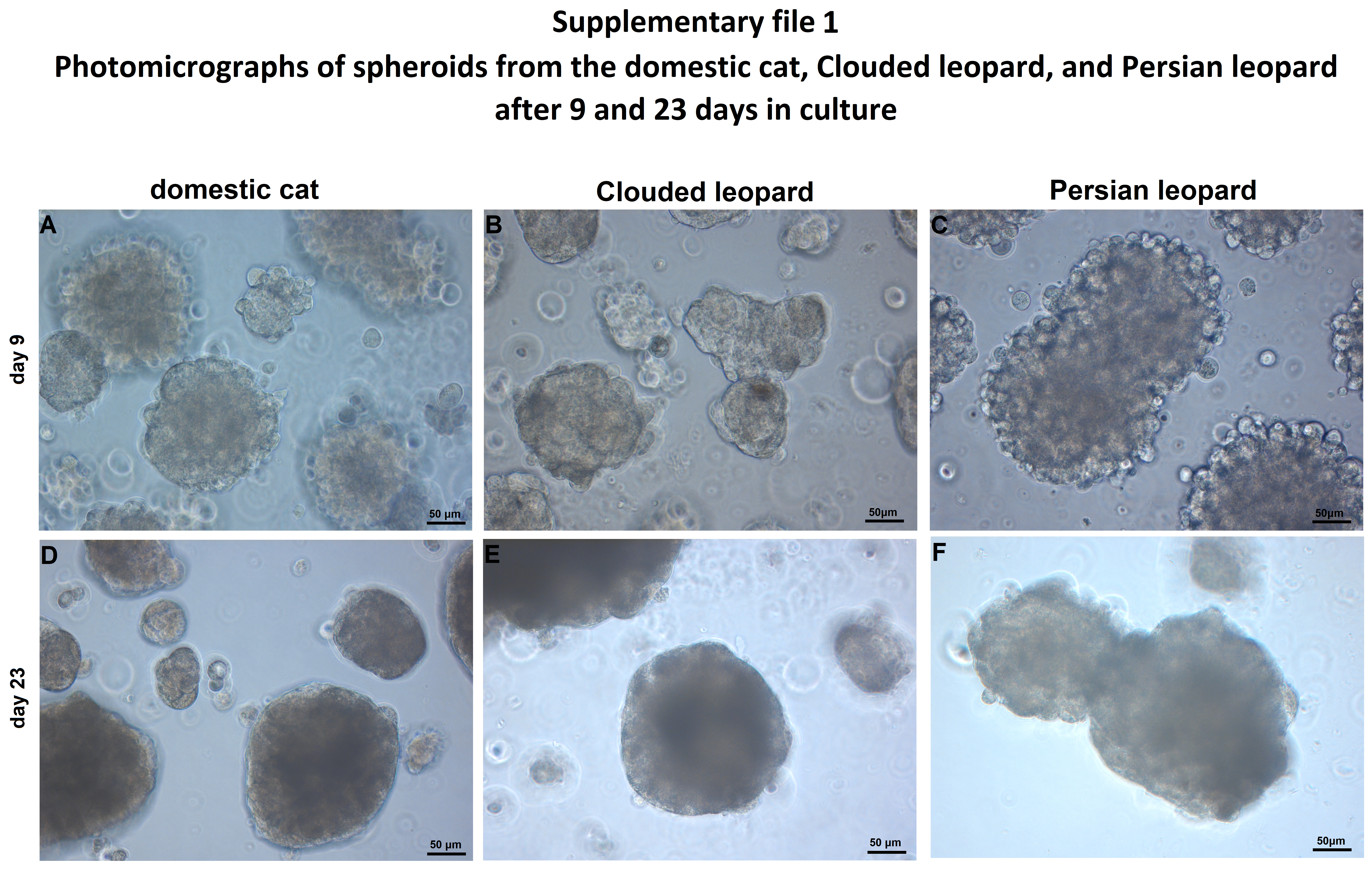

Supplement: Supplementary file 1 — Supplementary file1 Photomicrographs of spheroids from the domestic cat, Clouded leopard, and Persian leopard after 9 and 23 days in culture, respectively. (PNG 29.0 KB) [file 441_2024_3937_MOESM1_ESM.png]

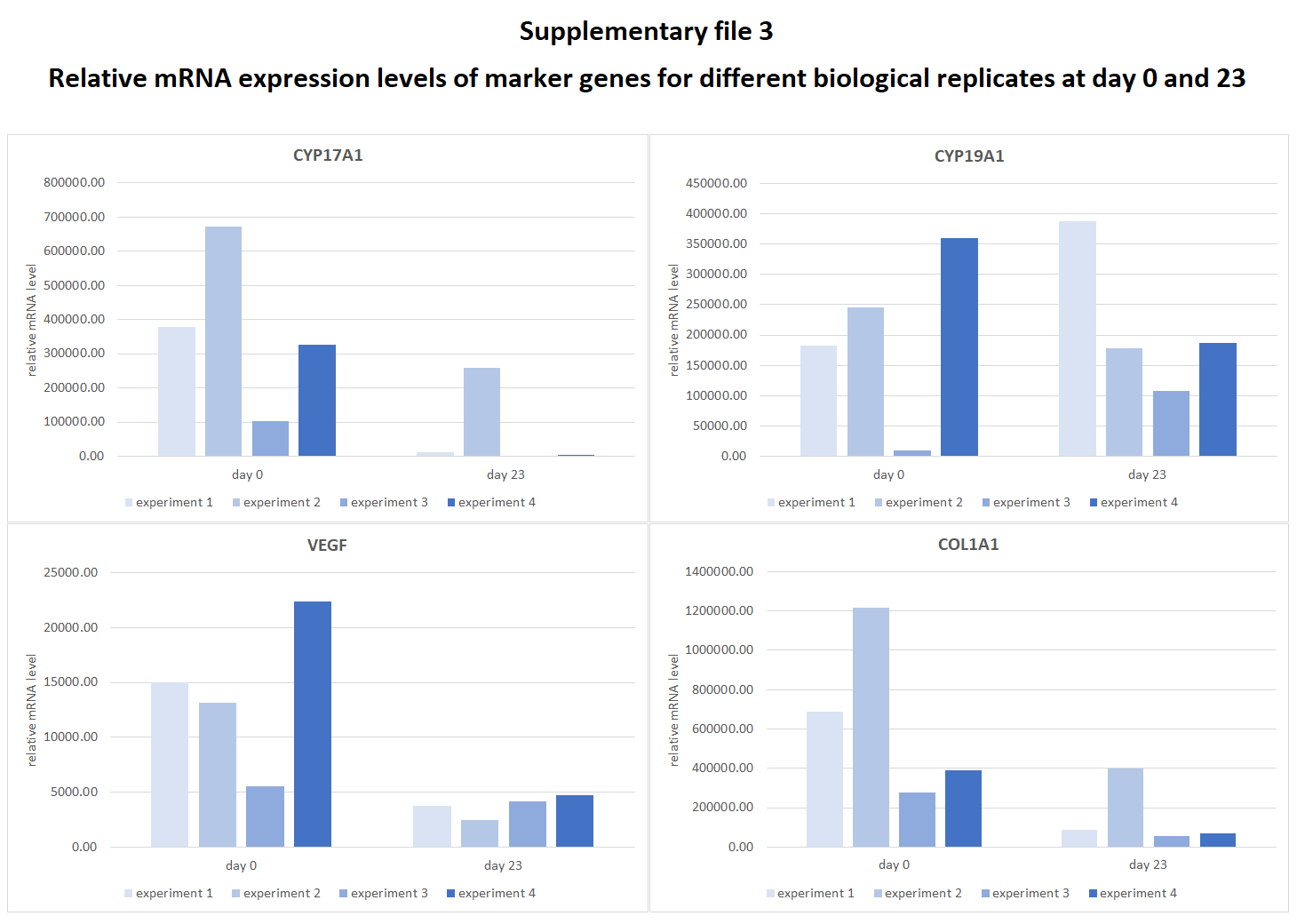

Supplement: Supplementary file 3 — Supplementary file3 Relative mRNA expression levels of marker genes for different biological replicates at day 0 and 23. (PNG 47.1 KB) [file 441_2024_3937_MOESM3_ESM.png]
