# Supplementary material for: Development of a 3D in vitro model to study corpus luteum of felids based on luteinized cells from antral follicles
Source: Cell Tissue Res. 2024 Dec 19;399(2):211–29. doi: 10.1007/s00441-024-03937-z (PMC11787223; doi:10.1007/s00441-024-03937-z)
Supplement: Supplementary file 2 — Supplementary file2 Preliminary experiment - comparison of steroidogenic activity of spheroids composed of different cell types. (DOCX 25.2 KB) [file 441_2024_3937_MOESM2_ESM.docx]

## Supplementary file 2

**Preliminary experiment - comparison of steroidogenic activity of spheroids composed of different cell types**

# Aim

The aim of the preliminary experiment was to compare steroidogenic activity (by measuring P4) of spheroids composed of different types of cells, based on isolation methods designed for different cell types.

# Material

Ovaries used for cells isolation come from the same sources as described in the main part of the article.

# Cell isolation

**Follicle cells mixture from antral follicles isolation:** as described in the main part of the article.

**Theca cells isolation:** closely follows the procedure used for the isolation of a mixed cells from antral follicles. Shortly after the antral follicles were isolated, granulosa cells and oocytes were released by incising the follicles and subsequently discarded. Residual GCs were mechanically removed using a scalpel, and the follicular lining was washed twice in Dulbecco's Phosphate-Buffered Saline to eliminate any remaining granulosa cells. The cleaned follicular lining was then subjected to further isolation steps using the same method described for follicular cell isolation. This approach aligns with the methodology for theca cell isolation described by Ma and Hao (Ma and Hao, 2018).

**Granulosa cells isolation:** granulosa cells were collected as previously described (Hryciuk et al., 2023).

# Cell culture

# The procedures for cell thawing, culture conditions, medium composition, and experimental setups were consistent with those outlined in the main section of the article. Granulosa cells, theca cells, and antral follicle cell mixtures were pooled together from five, three, and three animals, respectively. Cell viability after thawing of the pooled samples were as follows: granulosa cells – 78%, theca cells – 82%, follicle cells mixture – 73%.

# Cell suspensions were prepared by mixing the desired number of cells in Eppendorf tubes to achieve a concentration of 75,000 cells/mL. These suspensions were then distributed into culture wells, with each well containing 15,000 cells in 200 µL of medium.

# For each experimental group, three technical replicates (wells with cells) were prepared. The cell culture period extended over 14 days, with partial medium changes performed on days 2, 4, 7, 9, 11, and 14.

# Progestagen (P4) extraction and EIA

Hormone extraction and EIA measurements were performed similar as described in the main experiment. Progestagen (P4) concentration was measured in a medium collected at 2, 7 and 14. Progestagens concentration is reported as the amount of hormone produced per day. The measured values account for the hormone levels remaining after partial medium changes.

# Statistics

Statistical analysis was not performed, because the experiment was performed once.

# Results - Hormone concentration

Progestagens were detected across all experimental groups (Figure 1). On day 2, the highest progestagen concentration was observed in the following order: follicle cell mixture; granulosa cells; theca cells. This ranking in progestagen production, with the highest values listed first, was consistent on days 7 and 14 too. Notably, on day 14, the progestagen concentration in the follicle cell mixture group was considerably higher than in the other groups, e.g. being more than 40 fold greater than in the granulosa cell group and 83 fold greater than in the TC group.

Figure 1

# Conclusion

Based on the results obtained, the cell culture model utilizing follicular cell mixture appears to be the most effective for expressing a high steroidogenic activity. This model demonstrated a markedly higher progestagen production on day 7 and day 23 than the spheroids of other cell origin.

Hryciuk, M.M., Schröter, F., Hennicke, L., and Braun, B.C. (2023). Spheroid formation and luteinization of granulosa cells of felids in a long-term 3D culture. Differentiation; research in biological diversity *131*, 38-48.

Ma, Z., and Hao, C. (2018). Isolation of theca cells from whole ovary tissues may not be a suitable method. The Journal of biological chemistry *293*, 16174.
